# Associação entre Rigidez Arterial e Maior Densidade de Arritmia Atrial em Idosos Hipertensos sem Fibrilação Atrial

**DOI:** 10.36660/abc.20240251

**Published:** 2024-10-08

**Authors:** João Gabriel B. Lage, Alexandre L. Bortolotto, Luiz A. Bortolotto, Renata G. S. Verardino, Gabrielle D. Pessente, David C. S. Le Bihan, Rodrigo B. M. Barretto, Fernanda M. Consolim-Colombo, Denise T. Hachul, Luciana Sacilotto, Tan C. Wu, Sávia C. P. Bueno, Esteban W. R. Rivarola, César J. Gruppi, Silvio A. Barbosa, Juliana B. S. Alves, Wilson Mathias, Maurício I. Scanavacca, Francisco C.C. Darrieux

**Affiliations:** 1 Universidade de São Paulo Faculdade de Medicina Hospital das Clínicas São Paulo SP Brasil Unidade Clínica de Arritmia – Instituto do Coração (InCor), Hospital das Clínicas (HCFMUSP), Faculdade de Medicina, Universidade de São Paulo, São Paulo, SP – Brasil; 2 Universidade de São Paulo Faculdade de Medicina Hospital das Clinicas HCFMUSP São Paulo SP Brasil Unidade de Monitorização Ambulatorial – Instituto do Coração (InCor), Hospital das Clinicas HCFMUSP, Faculdade de Medicina, Universidade de São Paulo, São Paulo, SP – Brasil; 3 Universidade de São Paulo Faculdade de Medicina Hospital das Clinicas HCFMUSP São Paulo SP Brasil Unidade de Ecocardiografia – Instituto do Coração (InCor), Hospital das Clinicas HCFMUSP, Faculdade de Medicina, Universidade de São Paulo, São Paulo, SP – Brasil; 4 Universidade de São Paulo Faculdade de Medicina Hospital das Clinicas HCFMUSP São Paulo SP Brasil Unidade Clínica de Hipertensão – Instituto do Coração (InCor), Hospital das Clinicas HCFMUSP, Faculdade de Medicina, Universidade de São Paulo, São Paulo, SP – Brasil

**Keywords:** Rigidez Vascular, Cardiomiopatias, Função do Átrio Esquerdo

## Abstract

**Fundamento:**

A rigidez arterial aumentada é considerada atualmente um fator de risco independente para fibrilação atrial. No entanto, os mecanismos fisiopatológicos dessa arritmia ainda constituem uma lacuna no conhecimento a ser explorada.

**Objetivos:**

Investigar a existência de uma associação entre rigidez arterial e densidade de extrassístoles atriais em indivíduos hipertensos sem fibrilação atrial.

**Métodos:**

Estudo transversal com pacientes hipertensos sem fibrilação atrial diagnosticada, que foram estudados com ecocardiografia speckle-tracking para avaliar o strain do átrio esquerdo e velocidade de onda de pulso carótido-femoral (VOPcf) para avaliar a rigidez arterial. Todos os pacientes foram submetidos ao Holter de 24 horas e exames laboratoriais. O nível de significância adotado foi de p<0,05.

**Resultados:**

Setenta pacientes de um único centro sem doença cardiovascular evidente foram incluídos. A VOPcf correlacionou-se com uma maior densidade de extrassístoles atriais no Holter de 24 horas, independentemente da massa ventricular esquerda [1,48 (1,08- 2,03), p = 0,005]. Uma VOPcf aumentada correlacionou-se com valores reduzidos de strain atrial esquerdo, com coeficientes de correlação de Spearman de −0,27 (p= 0,027) e −0,29 (p = 0,018) para *strains* bidimensionais de reservatório e de conduto, respectivamente.

**Conclusão:**

Neste estudo com pacientes hipertensos, foi possível demonstrar uma associação entre rigidez arterial e maior densidade de arritmias atriais. Além disso, a rigidez arterial associou-se com valores mais baixos de strain atrial esquerdo das funções de reservatório e de conduto.

## Graphical abstract

**Figure:**
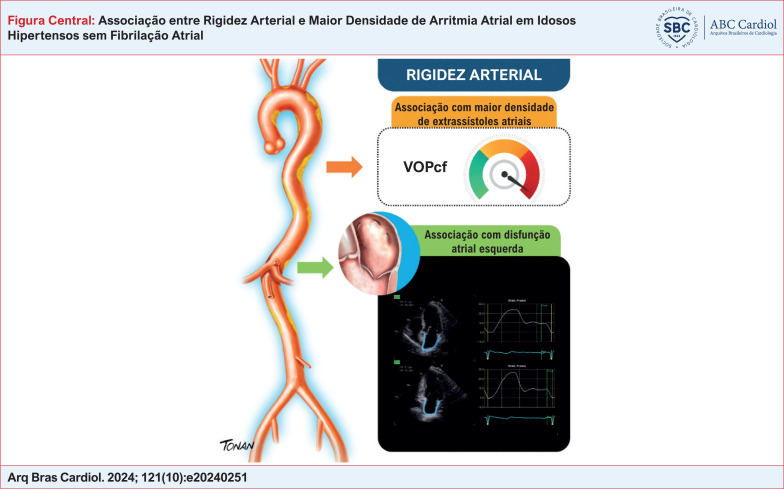
A rigidez arterial está associada com maior densidade de extrassístoles atriais e piora na função atrial esquerda (conduto e reservatório) mesmo antes da dilatação da cavidade; VOPcf: Velocidade de Onda de Pulso carótido-femoral.

## Introdução

A rigidez arterial é reconhecida como um marcador do desenvolvimento de doença cardiovascular.^1–4^ Ela pode ser avaliada por técnicas não invasivas, reprodutíveis e de baixo custo tais como a velocidade de onda de pulso (VOP), pressão de pulso, e *Augmentation Index*.^1^ Vários estudos mostraram que esses exames não invasivos para avaliar a rigidez arterial foram capazes de predizer eventos cardiovascular e mortalidade.^2–4^ A literatura atual mostra uma forte associação entre rigidez arterial e a incidência de fibrilação atrial (FA).^2,3,5,6^

O aumento do átrio esquerdo (AE) também é um fator de risco forte e independente para o desenvolvimento de FA e é parcialmente reversível devido à fibrose intersticial e alterações nos miócitos atriais.^7,8^ Os exames de ecocardiograma com *speckle-tracking* e o ecocardiograma tridimensional permitem a quantificação e a detecção de mudanças sutis na função do AE, em particular as propriedades de reservatório, conduto e contração do AE.^9,10^ A disfunção do AE, especialmente de reservatório e conduto, precede a dilatação do AE e aumenta o risco de desenvolvimento e recorrência da FA, independentemente de dilatação do AE.^10–14^

Apesar de inúmeras publicações descrevendo a associação da rigidez arterial e função do AE com a presença de FA, em nosso conhecimento, não há estudos sobre a associação entre essas medidas e estágios mais iniciais da cardiomiopatia atrial antes do desenvolvimento do fenótipo clínico propriamente dito da FA.

O objetivo deste estudo foi investigar a existência de uma associação da rigidez arterial e a função do AE analisada por ecocardiograma com *speckle-tracking* com a densidade de extrassístoles atriais em indivíduos hipertensos sem FA.

## Métodos

### População do estudo

A população do estudo foi recrutada no Instituto do Coração (InCor) do Hospital das Clínicas da Faculdade de Medicina da Universidade de São Paulo (HCFMUSP). Os pacientes com um diagnóstico de hipertensão e idade superior a 50 anos foram elegíveis a participar do estudo. Os pacientes foram selecionados consecutivamente no ambulatório de Hipertensão e Arritmia. Após a seleção, os pacientes foram submetidos a um Holter de 24 horas, um ecocardiograma, e medidas de VOP.

Para evitar possíveis fatores de confusão, os pacientes foram excluídos se apresentassem qualquer das seguintes condições: FA, hipertensão arterial secundária, diabetes mellitus com hemoglobina glicada (HbA1C) ≥ 9%; doença renal crônica com taxa de filtração glomerular (TFG) <45 mL/min (segundo fórmula do estudo MDRD - *Modification of Diet in Renal Disease study equation*); doenças da tireoide não controladas; pressão arterial sistólica (PAS) ≥ 160 mmHg e/ou pressão arterial diastólica (PAD) ≥100 mmHg; classe funcional II, III ou IV segundo a *Canadian Cardiovascular Society*; síndrome coronariana aguda por menos de um ano; insuficiência cardíaca; doenças valvares anatomicamente importantes; consumo de álcool ou drogas; uso de marcapasso, cardioversor desfibrilador implantável ou terapia de ressincronização cardíaca; presença de cardiopatia congênita; acidente vascular cerebral prévio; síndrome da apneia obstrutiva do sono; hipertrofia ventricular esquerda (VE) grave segundo a *American Society of Echocardiography* ou presença de distúrbios eletrolíticos de potássio, cálcio e magnésio.

O estudo foi conduzido de acordo com a Declaração de Helsinki e aprovado pela Comissão de Ética para Análise de Projetos de Pesquisa do pelo Comitê de Ética (CAPPesq) do HCFMUSP (SDC 4839/19/058) sob o parecer número 3.689.322. Todos os participantes assinaram um termo de consentimento, e concordaram em participar dos exames listados no projeto.

### Holter 24 horas

O exame foi realizado usando um aparelho com sistema de monitoramento com três canais de gravador CardioLight (Cardios®), e a análise realizada no CardioSmart (Cardios®).

### Fatores de risco

Os fatores de risco foram avaliados diretamente a partir dos prontuários médicos dos pacientes e por uma entrevista conduzida por um pesquisador treinado no dia dos testes. Hipertensão foi definida como PAS igual ou superior a 140 mmHg ou PAD igual ou superior a 90 mmHg, ou pelo uso de medicamentos anti-hipertensivos. Diabetes mellitus foi definido por glicemia de jejum igual ou superior a 126 mg/dL ou uso atual de insulina ou agentes hipoglicemiantes. O índice de massa corporal (IMC) foi calculado dividindo-se o peso (Kg) pela altura ao quadrado (m^2^). Tabagista atual foi definido como uma pessoa que fumou mais de 100 cigarros ao longo da vida e ter fumado um ou mais cigarros diariamente no momento da entrevista. Um ex-tabagista foi definido como uma pessoa que fumou mais de 100 cigarros por dia ao longo da vida, mas relatou que parou de fumar. A TFG foi estimada usando a fórmula do MDRD.

### Medida da velocidade de onda de pulso

A VOP foi avaliada no segmento arterial carótido-femoral (cf) (VOP carótido-femoral, VOPcf) usando o aparelho Complior® (ALAM Medical, Vincennes, França). A medida foi feita pelo posicionamento simultâneo de dois sensores mecanográficos nas artérias carótida e femoral localizadas a uma distância conhecida. Esses sensores contêm membranas que são deformadas sucessivamente pelo choque da onda de pulso e tal deformação é inicialmente transformada em sinal elétrico, e posteriormente transmitida para um programa de computador para o cálculo.

A medida da VOPcf foi realizada com o paciente na posição supina, após 10 minutos de descanso, em um ambiente silencioso, com temperatura controlada. A distância entre a artéria carótida e a artéria femoral foi medida por uma fita métrica, nos pontos em que os transdutores foram colocados. Essa informação foi inserida diretamente no software.

Cada onda de pulso aparece em tempo real na tela do computador e o aparelho determina a fase ascendente inicial da onda e o início da onda em dois pontos diferentes, deduzindo a velocidade como uma função da distância conhecida. Após análise de 10 curvas, a velocidade média foi registrada.

### Exame ecocardiográfico

Exames de ecocardiograma transtorácico foram agendados para todos os pacientes. Um aparelho GE® (Vivid E95, EUA) foi usado, com transdutores de múltipla frequência. Medidas cardíacas volumétricas foram adquiridas por exames ecocardiográficos convencionais, ecocardiograma com doppler tecidual, bidimensional, tridimensional e em modo M. A aquisição e a análise das imagens foram realizadas seguindo-se as diretrizes da *American Society of Echocardiography* (ASE). Para cada variável ecocardiográfica, foram analisados pelo menos três ciclos. Os exames foram avaliados por um investigador experiente.

**Figura 1:**
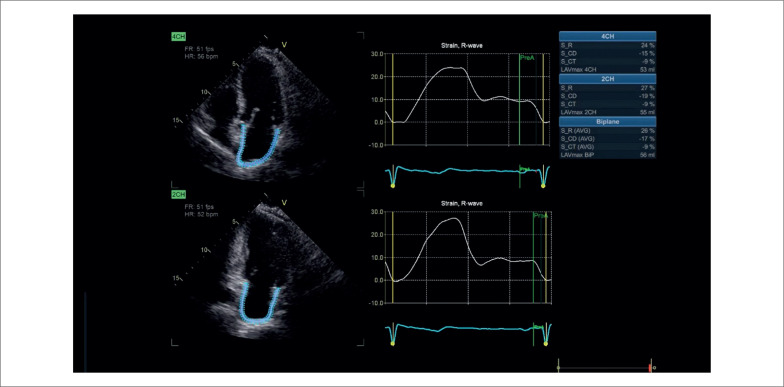
Análise da função atrial esquerda por ecocardiograma bidimensional com *speckle-tracking*.

A função do AE foi avaliada por medidas de *strain* bidimensional obtidas por ecocardiograma com *speckle-tracking*, usando o programa AFI LA embutido na *workstation* EchoPAC PC versão 204 (GE Healthcare, Horten, Noruega) (Figura 1).

**Figura 2:**
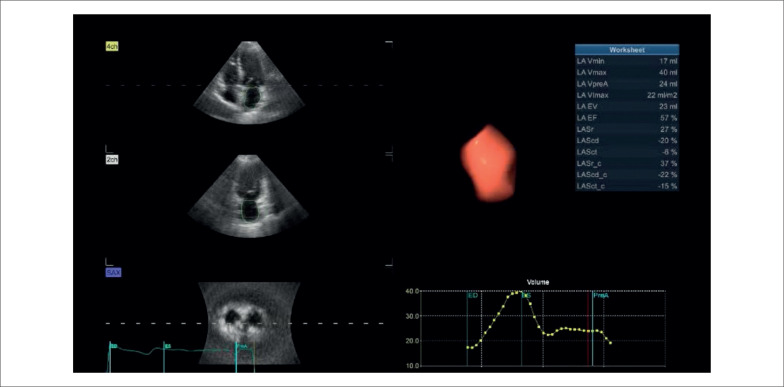
Análise da função atrial esquerda por ecocardiograma tridimensional com *speckle-tracking*.

Para a avaliação da função do AE, utilizou-se a ecocardiografia tridimensional, com a aquisição completa de seis batimentos na janela apical. Utilizando-se o software 4D LAQ embutido na workstation EchoPAC PC workstation versão 204 (GE Healthcare, Horten, Noruega), foi possível calcular volumes do AE e fração de esvaziamento (Figura 2).

### Análise estatística

Para o cálculo amostral, considerou-se um número mínimo de 30 pacientes, com base em um poder de detectar alterações nas medidas de VOPcf em nosso serviço, de acordo com a reprodutibilidade e a sensibilidade do teste. O método apresentou coeficientes de reprodutibilidade de intraobservador e entre observadores de 0,935 e 0,890, respectivamente.

Para avaliar a normalidade da distribuição dos dados contínuos, o teste de Kolmogorov-Smirnov foi usado. Para as variáveis contínuas com distribuição normal, os resultados foram apresentados em média e desvio padrão, e para aquelas sem distribuição normal, os resultados são apresentados em mediana e intervalo interquartil. Para as variáveis categóricas, foram usadas frequências absolutas e relativas.

O coeficiente de correlação de Spearman foi usado para comparar a VOPcf com o índice de massa e o número de extrassístoles atriais em 24 horas, bem como comparar o índice de massa com o número de extrassístoles atriais. O coeficiente também foi usado para avaliar a correlação entre VOPcf com as variáveis de função do AE.

Um modelo linear generalizado com distribuição gama foi usado para determinar a associação independente entre VOPcf e outras variáveis de interesse.

Um valor de p<0,05 foi considerado estatisticamente significativo. Os cálculos foram realizados usando o programa R4.0.5 (R Core Team, 2021).

## Resultados

Um total de 3137 pacientes consecutivos foram selecionados inicialmente. Após excluir os pacientes que não preencheram os critérios de inclusão do estudo, 303 pacientes foram considerados elegíveis e, desses, 70 foram incluídos após assinarem um termo de consentimento. Com base nos critérios de exclusão, e pelo fato de nosso hospital universitário ser um centro de atenção terciária, em que a maioria dos pacientes atendidos têm múltiplas comorbidades, muitos pacientes não foram considerados elegíveis para evitar possíveis vieses de confusão. Parte do estudo foi desenvolvido durante a pandemia da COVID-19, em que os pacientes evitavam ir ao hospital para realizar exames.

As características gerais dos pacientes estão listadas na Tabela 1. A maioria dos pacientes eram idosos, do sexo feminino, e apresentaram síndrome metabólica. Os pacientes apresentaram uma mediana de 72 extrassístoles atriais por hora e função VE preservada, 41,4% da população era diabética, com HbA1C média de 6,19%.

**Tabela 1:** Características basais dos pacientes do estudo (n=70)

		Total (70)
**Sexo**		
	Feminino	48/70 (68,6%)
	Masculino	22/70 (31,4%)
**Etnia**		
	Afro-Brasileira	27/70 (38,6%)
	Caucasiana	43/70 (61,4%)
**Idade (anos)**		66,70 ± 7,83
**Peso (Kg)**		77,57 ± 16,61
**Altura (Cm)**		162,21 ± 9,68
**Circunferência abdominal (cm)**		103,09 ± 13,82
**IMC (Kg/m²)**		29,39 ± 5,44
**Tabagista**		7/70 (10,0%)
**Ex-tabagista**		10/70 (14,3%)
**IECA/BRA**		63/70 (90,0%)
**Betabloqueador**		39/70 (55,7%)
**BCC**		33/70 (47,1%)
**Diuréticos**		45/70 (64,3%)
**Estatinas**		50/70 (71,4%)
**Pressão de pulso (mmHg)**		48,87 ± 16,19
**Número de anti-hipertensivos**		2,50 [2,00; 3,00]
**Índice de massa ventricular esquerda (g/m²)**		82,63 ± 18,99
**Extrassístoles atriais por hora no Holter 24h**		72,00 [16,25; 510,25]
**TFG, MDRD (ml/min/1,73m²)**		76,18 ± 15,77
**Triglicerídeos (mg/dL)**		128,01 ± 62,65
**HDL (mg/dL)**		55,14 ± 15,03
**LDL (mg/dL)**		109,96 ± 36,60
**HbA1C (%)**		6,19 ± 0,92
**Diabetes mellitus**		29/70 (41,4%)
**PAS (mmHg)**		143,37 ± 18,83
**PAD (mmHg)**		83,64 ± 10,28
**Frequência cardíaca (bpm)**		71,26 ± 15,19
**FEVE (%)**		67,00 [64,00; 69,00]
**Volume atrial esquerdo indexado (mL/m²)**		31,96 ± 9,56
**Disfunção diastólica > Grau I**		10/70 (14,3%)
**Peptídeo natriurético cerebral >100 pg/mL**		9/69 (1,0%)
**Troponina >p99**		2/69 (2,9%)

IECA: inibidores da enzima conversora de angiotensina; BRA: bloqueadores do receptor da angiotensina II; IMC: índice de massa corporal; BCC: bloqueador dos canais de cálcio; TFG: Taxa de Filtração Glomerular; MDRD: equação do estudo *Modification of Diet in Renal Disease*; HbA1C: hemoglobina glicada ou hemoglobina A1C; HDL: lipoproteína de alta densidade; LDL: lipoproteína de baixa densidade; FEVE: fração de ejeção ventricular esquerda; PAS: pressão arterial sistólica; PAD: pressão arterial diastólica; PAS: pressão arterial sistólica.

Observou-se uma correlação significativa entre extrassístoles atriais em 24 horas e índice de massa VE (0,27; p=0,025). Após análise ajustada pela massa VE, utilizando um modelo linear generalizado com distribuição gama, observou-se uma associação positiva da VOPcf com extrassístoles atriais em 24 horas, independentemente da massa VE. Também se observou que, para cada aumento de uma unidade na VOPcf, houve uma associação com um aumento médio de 1,48 extrassístoles atriais no Holter de 24 horas (Tabela 2).

**Tabela 2:** Associação da Velocidade de onda de pulso carótido-femoral com extrassístoles atriais em 24 horas obtidas de um modelo linear generalizado com distribuição gama

	Coeficiente (IC de 95%)	Valor p
Intercepto	72,57 (0,68; 4982,16)	0,017
VOPcf (m/s)	1,48 (1,08; 2,03)	0,005
Índice de massa VE (g/m²)	0,99 (0,94; 1,03)	0,435

VOPcf: velocidade de onda de pulso carótido-femoral; VE: ventricular esquerda.

A Tabela 3 apresenta as correlações de Spearman da VOPcf com as diferentes variáveis da função do AE. É possível observar uma correlação negativa significativa com as medidas de *strain* bidimensional do AE nas funções de reservatório e de conduto. Ainda, uma correlação negativa foi observada para a fração de esvaziamento atrial passiva.

**Tabela 3:** Correlação de Spearman (valor p) da Velocidade de Onda de Pulso carótido-femoral com as respectivas medidas de *strain* do átrio esquerdo

			Correlação (valor p)
**Ecocardiograma bidimensional**			
	Fração de ejeção ventricular esquerda (%)		0,11 (0,375)
	Volume atrial esquerdo indexado (mL/m²)		-0,07 (0,589)
***Strain* bidimensional (%)**			
	Reservatório biplanar		-0,27 (0,027)
	Conduta biplanar		-0,29 (0,018)
	Contração biplanar		-0,12 (0,325)
	Volumes 3D (mL)		
		Máximo	0,02 (0,879)
		Mínimo	0,10 (0,422)
		Pre-A	0,12 (0,346)
**Fração de esvaziamento atrial 3D (%)**			
	Total		-0,22 (0,078)
	Ativo		0,00 (0,974)
	Passivo		-0,39 (0,002)
**Índice de distensibilidade atrial**			-0,22 (0,078)

## Discussão

### Rigidez arterial e densidade de arritmia atrial

Uma revisão recente^15^ relatou uma associação entre rigidez arterial e incidência de FA em diferentes populações, mesmo após procedimentos de cardioversão e ablação por cateter.^15^

Em geral, a origem das extrassístoles atriais não é fácil de ser demonstrada pelo Holter. Na presença de extrassístole atrial, como já se sabe, quanto mais novo o paciente (e ausência de comorbidades), maior a chance de origem da extrassístole atrial ser no átrio direito, sendo a maioria originária na crista terminalis. Já nos pacientes mais velhos e hipertensos, essa probabilidade é mais remota e o AE torna-se a fonte das extrassístoles atriais, principalmente com rigidez atrial ou sobrecarga atrial causada por hipertensão. Tivemos o cuidado de excluir taquicardias atriais e extrassístoles atriais de foco único pela análise dos traçados de Holter.

Encontrou-se uma correlação significativa entre a VOPcf e extrassístoles atriais em 24 horas. Também se observou uma correlação do índice de massa VE com extrassístoles atriais em 24 horas e VOPcf; uma vez que um índice de massa VE mais alto pode representar um fator de confusão,^16,17^ decidimos realizar um modelo linear generalizado com distribuição gama, ajustando para essa variável. A correlação entre VOPcf e extrassístoles atriais continuou significativa, indicando uma associação independente.

Esses resultados podem representar evidência inicial na literatura de uma associação entre rigidez arterial e arritmias atriais, anda na fase pré-fibrilatória, em indivíduos hipertensos, independentemente do índice de massa VE. Embora essa correlação tenha sido considerada fraca, o fato de existir plausibilidade biológica e que encontramos essa correlação com FA sugere fortemente que foi um achado ocasional.

### Função atrial esquerda

No presente estudo, pudemos demonstrar que quanto maior a VOP, menores foram os valores de *strain* bidimensional de reservatório e de conduto, bem como a fração de esvaziamento passivo do AE. Tais observações sugerem que a rigidez arterial pode primeiro afetar as funções de reservatório e de conduto, e que a dilatação ocorre posteriormente, à medida que a rigidez arterial aumenta, representando um marcador mais tardio do remodelamento atrial, como demonstrado por Yoshida et al.^18^ em um estudo similar com indivíduos com FA. É importante destacar que todos os pacientes estudados apresentaram índice de massa VE dentro dos valores de normalidade.

Nossos achados destacam a importância de se avaliar a função do AE em indivíduos com rigidez arterial aumentada, mesmo na ausência de uma doença cardiovascular clara. *Strain* atrial esquerdo, particularmente *strain* de reservatório e de conduto, foi descrito como um marcador diagnóstico mais sensível de disfunção diastólica VE que parâmetros convencionais de Doppler espectral e Doppler tecidual.^19,20^

Brecht et al.^21^ demonstraram que uma disfunção no *strain* de reservatório e de conduto foi associada com disfunção diastólica e que a alteração desses dois parâmetros precedeu dilatação do AE e/ou elevação do E/e’.^21^

### Importância clínica

Nossos dados são importantes para compreender a fisiopatologia da cardiomiopatia atrial, especialmente no que chamamos hoje de fase pré-fibrilatória. Embora a correlação entre VOP e densidade de ectopia atrial tenha sido fraca, o fato de que já há estudos demonstrando essa associação com FA reforça a plausibilidade biológica de nossos achados e sua concordância com a literatura atual. Ainda, reforça-se a importância da detecção precoce do remodelamento atrial nesses pacientes, mesmo antes da dilatação do AE, abrindo caminho para estratégias preventivas, para interromper a cascata de fibrose atrial, com o desenvolvimento, com consequente desenvolvimento de FA.

De fato, o benefício de se intervir nesta fase antes da dilatação atrial já foi demonstrado. Kokubu et al.^20^ relataram uma melhora significativa no *strain* de reservatório em pacientes hipertensos sem dilatação do AE duas semanas após terapia com inibidor de enzima conversora de angiotensina, enquanto não se observou melhora em pacientes com aumento de AE.

### Limitações do estudo

Nosso estudo tem algumas limitações. O ecocardiograma e a medida de VOP devem ser realizados, idealmente, de maneira simultânea. No entanto, uma vez que a técnica ecocardiográfica não permite medida simultânea da VOP, os testes não puderam ser realizados ao mesmo tempo.

O pequeno tamanho amostral pode ser explicado pelo fato de nosso hospital ser um centro de referência terciário, com uma proporção menor de pacientes com hipertensão arterial sem outras comorbidades, uma vez que esses pacientes são geralmente atendidos em serviços de atenção primária em nosso país. Outra possível limitação é a possibilidade de que todos esses pacientes tenham apresentado episódios de FA excluídos por instrumentos mais precisos, como um monitor cardíaco implantável. Apesar disso, essa seleção de pacientes realmente reflete a prática clínica do "mundo real".

Finalmente, é importante enfatizar que este é um estudo observacional transversal. Assim, não é possível estabelecer uma relação de causa e efeito, e outros estudos prospectivos do tipo coorte são necessários nesta área.

## Conclusão

Com base em nossas observações, concluímos que a rigidez arterial associou-se com uma maior carga de extrassístoles atriais, com piora da função do AE (conduto e reservatório), mesmo antes da dilatação dessa cavidade nos pacientes idosos hipertensos avaliados (Figura Central).

Mais estudos com um número maior de pacientes são necessários para corroborar nossos achados, uma vez que uma associação entre rigidez arterial e uma maior densidade de extrassístoles atriais foi observada antes do início da FA.

Ensaios clínico futuros sobre hipertensão arterial devem analisar o impacto dos medicamentos, não só sobre a rigidez arterial como sobre a função do AE, e verificar os benefícios na redução do índice de massa do VE e melhora da função atrial, principalmente naqueles com extrassístoles atriais frequentes.

## *Material suplementar

Para informação adicional, por favor, clique aqui.
