# Supplementary material for: Associação entre Rigidez Arterial e Maior Densidade de Arritmia Atrial em Idosos Hipertensos sem Fibrilação Atrial
Source: Arq Bras Cardiol. 2024 Oct 8;121(10):e20240251. [Article in Portuguese] doi: 10.36660/abc.20240251 (PMC11634220; doi:10.36660/abc.20240251)
Supplement: Translation

# Translation: Association between Arterial Stiffness and Higher Burden of Atrial Arrhythmia in Elderly Hypertensive Patients without Atrial Fibrillation

**DOI:** 10.36660/abc.20240251i

**Authors:** João Gabriel B. Lage, Alexandre L. Bortolotto, Luiz A. Bortolotto, Renata G. S. Verardino, Gabrielle D. Pessente, David C. S. Le Bihan, Rodrigo B. M. Barretto, Fernanda M. Consolim-Colombo, Denise T. Hachul, Luciana Sacilotto, Tan C. Wu, Sávia C. P. Bueno, Esteban W. R. Rivarola, César J. Gruppi, Silvio A. Barbosa, Juliana B. S. Alves, Wilson Mathias, Maurício I. Scanavacca, Francisco C.C. Darrieux

**Affiliations:** 1 São Paulo SP Brazil Unidade Clínica de Arritmia – Instituto do Coração (InCor), Hospital das Clínicas (HCFMUSP), Faculdade de Medicina, Universidade de São Paulo, São Paulo, SP – Brazil; 2 São Paulo SP Brazil Unidade Clínica de Monitorização Ambulatorial – Instituto do Coração (InCor), Hospital das Clinicas HCFMUSP, Faculdade de Medicina, Universidade de São Paulo, São Paulo, SP – Brazil; 3 São Paulo SP Brazil Unidade de Ecocardiografia – Instituto do Coração (InCor), Hospital das Clinicas HCFMUSP, Faculdade de Medicina, Universidade de São Paulo, São Paulo, SP – Brazil; 4 São Paulo SP Brazil Unidade Clínica de Hipertensão – Instituto do Coração (InCor), Hospital das Clinicas HCFMUSP, Faculdade de Medicina, Universidade de São Paulo, São Paulo, SP – Brazil

**Keywords:** Vascular Stiffness, Cardiomyopathies, Left Atrial Function

## Abstract

**Background:**

Increased arterial stiffness is currently an independent risk factor for atrial fibrillation, but the pathophysiological mechanisms of this arrhythmia remain an area of knowledge gap to be explored.

**Objectives:**

To investigate the existence of an association between arterial stiffness and the density of premature atrial contractions (PACs) in hypertensive individuals without atrial fibrillation.

**Methods:**

Cross-sectional study with hypertensive patients without diagnosed atrial fibrillation, who were studied with speckle-tracking echocardiography to assess left atrial (LA) strain and carotid-femoral pulse wave velocity (cfPWV) to assess arterial stiffness. All patients underwent 24h-ECG Holter and laboratory tests. Significance level was set at p<0.05.

**Results:**

Seventy participants from a single centre without overt cardiovascular disease were included. The cfPWV was correlated with higher density of PACs in 24h-Holter monitoring, independently of LV mass index (1.48 [1.08-2.03], p-value 0.005). Increased cfPWV was correlated with decreased LA strain values, with Spearman correlation coefficients of −0.27 (p-value 0.027) and −0.29 (p-value 0.018) for reservoir and conduit 2D Strain, respectively.

**Conclusions:**

In this study with hypertensive patients, it was possible to demonstrate an association between arterial stiffness and higher density of atrial arrhythmias. Furthermore, arterial stiffness was associated with lower left atrial strain values for reservoir and conduit functions.

## Introduction

Arterial stiffness is recognized as a marker for the development of cardiovascular disease.^1–4^ It can be assessed with non-invasive, reproducible, and inexpensive techniques such as pulse wave velocity (PWV), central pulse pressure, and augmentation index.^1^ Several studies have shown that these non-invasive arterial stiffness tests were able to predict cardiovascular events and mortality.^2–4^ The current literature shows a strong association between arterial stiffness and the incidence of atrial fibrillation (AF).^2,3,5,6^

Left atrial (LA) enlargement is also a strong and independent risk factor for AF^7^ and is partially reversible due to interstitial fibrosis and changes in atrial myocytes.^7,8^ Speckle-tracking echocardiography (STE) and three-dimensional echocardiography allow the quantification and detection of subtle changes in left atrial function, particularly reservoir, conduit, and contraction LA properties.^9,10^ LA dysfunction, especially reservoir and conduit dysfunction, precedes LA dilation and increases the risk of development and recurrence of AF, independent of LA dilation.^10–14^

Despite extensive publications describing the association of arterial stiffness and LA function with the presence of AF, to the best of our knowledge, there are no studies on the association between these measurements and earlier stages of atrial cardiomyopathy before the development of AF clinical phenotype.

The aim of this study is to investigate the existence of an association between arterial stiffness and LA function analysis by STE with the density of premature atrial contractions (PACs) in hypertensive individuals without AF.

## Methods

### Study population

The study population was recruited at InCor – Heart Institute of the University of São Paulo Medical School. Patients with a diagnosis of hypertension and aged over 50 years old were eligible to participate in the study. Patients were consecutively selected from the Hypertension and Arrhythmia Outpatient Clinic. After selection, such patients underwent a 24h-Holter monitoring, were studied with echocardiography, and underwent pulse wave velocity measurements.

In order to avoid possible confounding factors, patients were excluded for any of the following reasons: AF; secondary arterial hypertension; diabetes mellitus with glycated hemoglobin (HbA1C) ≥ 9%; chronic kidney disease with glomerular filtration rate (GFR) <45 mL/min (MDRD – Modification of Diet in Renal Disease study equation); uncontrolled thyroid disorders; systolic blood pressure (SBP) ≥160 mmHg and/or diastolic blood pressure (DBP) ≥100 mmHg; Canadian Cardiovascular Society functional classes II, III or IV; acute coronary syndrome for less than one year; heart failure; anatomically important valve diseases; alcoholism or drug use; presence of a pacemaker, implantable cardioverter defibrillator or cardiac resynchronization therapy; presence of residual congenital heart disease; previous stroke; obstructive sleep apnea syndrome; severe left ventricular (LV) hypertrophy, according with the American Society of Echocardiography or the presence of electrolyte imbalances of potassium, calcium and magnesium.

The study was conducted according to the Declaration of Helsinki and was approved by the Ethics Committee for the Analysis of Research Projects (CAPPesq) of HCFMUSP (SDC 4839/19/058) under approval number 3.689.322. All participating individuals signed an informed consent form, agreeing to undergo the exams listed in the project.

### 24-hour ECG recording (Holter monitoring)

The exam was performed using a 3-channel Digital Recorder – CardioLight (Cardios®), with analysis performed in CardioSmart (Cardios®).

### Risk factor assessment

Risk factors were checked directly from the patient's medical record and through an interview conducted by a trained researcher on the day of the tests. Hypertension was defined as SBP of at least 140 mmHg or DBP of at least 90 mmHg, or the use of antihypertensive medications. Diabetes mellitus was defined by a fasting blood glucose level of at least 126 mg/dL or current use of insulin or oral hypoglycemic agents. Body mass index was calculated as weight (Kg) divided by height squared (m²). A current smoker was defined as having smoked more than 100 cigarettes in one's lifetime and having smoked at least one cigarette daily at the time of the interview. A former smoker was someone who had smoked more than 100 cigarettes in their lifetime but reported that they had quit smoking. The GFR was estimated MDRD.

### Measurement of pulse wave velocity (PWV)

PWV was assessed in the carotid-femoral (cf) arterial segment using the Complior® device (ALAM Medical, Vincennes, France). The measurement was made by means of simultaneous positioning of two mechanographic sensors in the carotid and femoral arteries located at a known distance. These sensors contain membranes that are successively deformed by the shock of the pulse wave and this deformation is initially transformed into an electrical signal and later transmitted to a calculation computer program.

The cfPWV test was performed with the patient in the supine position, after 10 minutes of rest, in a quiet, temperature-controlled environment. The distance between the carotid artery and the femoral artery was measured using a tape measure, at the points where the transducers were placed. This information has been entered directly into the software.

Each pulse wave appears in real time on the computer screen and the device determines the initial ascending phase of the wave and the beginning of the wave at two different points, inferring the speed as a function of the known and previously entered distance. After 10 curves followed by analysis, the mean value was recorded.

### Echocardiographic examination

Transthoracic echocardiograms were scheduled for all patients. A GE® (Vivid E95, USA) echocardiography machine was used, with matrixial multifrequency and volumetric transducers and resources to obtain the modalities of M-mode, two and three-dimensional, tissue Doppler, and conventional Doppler echocardiography.. American Society of Echocardiography (ASE) guidelines were followed for image acquisition and analysis. For each echocardiographic variable, at least three cardiac cycles were analyzed. The exams were evaluated by the same experienced investigator.

LA function was evaluated by means of two-dimensional strain measurements obtained by STE, using the software AFI LA embedded into the EchoPAC PC workstation version 204 (GE Healthcare, Horten, Norway) (Figure 1).

**Figure 1:** Analysis of left atrial function by 2D speckle-tracking echocardiography.

For the evaluation of LA function using three-dimensional (3D) echocardiography, with six-beat full-volume acquisitions in the apical windows. Using the 4D LAQ software embedded in the EchoPAC PC workstation version 204 (GE Healthcare, Horten, Norway), it was possible to calculate LA volumes and emptying fractions (Figure 2)

**Figure 2:** Analysis of left atrial function by 3D speckle-tracking echocardiography.

### Statistical analysis

For sample calculation, a minimum number of 30 patients was considered, based on the power to detect alterations in cfPWV measurements observed in our center, according to the reproducibility and sensitivity of the test. The method had intra-observer and inter-observer reproducibility coefficients of 0.935 and 0.890, respectively.

To assess the assumption of normality of continuous data, the Kolmogorov-Smirnov test was used. For continuous variables with normal distribution, results are presented as mean ± standard deviation, and for those without a normal distribution, results are presented as median and interquartile range. For categorical variables, absolute and relative frequencies were used.

Spearman's correlation coefficient was used to compare the cfPWV with the mass index and the number of PACs in 24h, as well as to compare the latter two. Besides, it was also used to evaluate the correlation between cfPWV with LA function variables.

A generalized linear model with gamma distribution was used to determine the independent association between cfPWV and other variables of interest.

A p-value <0.05 was considered statistically significant. Calculations were performed using the R 4.0.5 software (R Core Team, 2021).

## Results

A total of 3,137 consecutive patients were initially selected. After excluding patients who did not meet the study inclusion criteria, 303 patients were eligible and, of these, 70 were included after signing the consent form. Based on the exclusion criteria, and because our university hospital is a tertiary centre, where most patients have multiple comorbidities, many patients were not eligible to avoid possible confounding bias; part of the study was performed during the COVID-19 pandemic, where patients were avoiding coming to the hospital for exams.

General characteristics of the patients are listed in Table 1. Most patients were older, female individuals, with metabolic syndrome. They had a median of 72 PACs per hour and preserved LV function; 41.4% of the population was diabetic, with an average HbA1C of 6.19%.

**Table 1:** Baseline characteristics of the study patients (n=70)

		Total (70)
**Sex**		
	Female	48/70 (68.6%)
	Male	22/70 (31.4%)
**Ethnicity**		
	Afro-Brazilian	27/70 (38.6%)
	Caucasian	43/70 (61.4%)
**Age (Years)**		66.70 ± 7.83
**Weight (Kg)**		77.57 ± 16.61
**Height (cm)**		162.21 ± 9.68
**Abdominal circumference (cm)**		103.09 ± 13.82
**BMI (Kg/m²)**		29.39 ± 5.44
**Smoker**		7/70 (10.0%)
**Former smoker**		10/70 (14.3%)
**ACEi/ARB**		63/70 (90.0%)
**Beta-blocker**		39/70 (55.7%)
**CCB**		33/70 (47.1%)
**Diuretic**		45/70 (64.3%)
**Statins**		50/70 (71.4%)
**Central pulse pressure (mmHg)**		48.87 ± 16.19
**Number of antihypertensives**		2.50 [2.00; 3.00]
**LV mass index (g/m²)**		82.63 ± 18.99
**Premature atrial contractions per hour in 24h-Holter**		72.00 [16.25; 510.25]
**GFR MDRD (ml/min/1,73m²)**		76.18 ± 15.77
**Triglycerides (mg/dL)**		128.01 ± 62.65
**HDL (mg/dL)**		55.14 ± 15.03
**LDL (mg/dL)**		109.96 ± 36.60
**HbA1C (%)**		6.19 ± 0.92
**Diabetes mellitus**		29/70 (41.4%)
**SBP (mmHg)**		143.37 ± 18.83
**DBP (mmHg)**		83.64 ± 10.28
**Heart rate (bpm)**		71.26 ± 15.19
**LVEF (%)**		67.00 [64.00; 69.00]
**Indexed LA volume (ml/m²)**		31.96 ± 9.56
**Diastolic dysfunction > Grade I**		10/70 (14.3%)
**BNP >100 pg/mL**		9/69 (1.0%)
**Troponin >p99**		2/69 (2.9%)

ACEi: angiotensin converting enzyme inhibitors; ARB: angiotensin II receptor blockers; BMI: body mass index; BNP: brain natriuretic peptide; CCB: calcium channel blockers; DBP: diastolic blood pressure; GFR MDRD: glomerular filtration rate by the Modification of Diet in Renal Disease study equation; HbA1C: glycosylated hemoglobin or hemoglobin A1C; HDL: high-density lipoprotein cholesterol; LA: left atrial; LDL: low-density lipoprotein cholesterol; LV: left ventricular; LVEF: left ventricular ejection fraction; SBP: systolic blood pressure.

A significant correlation was observed between PACs in 24h and LV mass index (0.27, p= 0.025). After analysis adjusted by LV mass, using a generalized linear model with gamma distribution, a positive association of cfPWV with PACs in 24 hours was still observed, independently of LV mass. It was also found that for each increase of one unit in cfPWV there was an association with an average increase of 1.48 PACs in the 24h-Holter monitoring analysis (Table 2).

**Table 2:** Results of the association of carotid-femoral pulse wave velocity and left ventricular mass index with premature atrial contractions in 24h obtained from a generalized linear model with gamma distribution

	Coefficient (95% CI)	p-value
Intercept	72.57 (0.68; 4982.16)	0.017
cfPWV (m/s)	1.48 (1.08; 2.03)	0.005
LV mass index (g/m²)	0.99 (0.94; 1.03)	0.435

cfPWV: carotid-femoral pulse wave velocity; LV: left ventricular.

Table 3 presents the Spearman correlations of cfPWV with the different LA function variables. It is possible to observe a significant negative correlation with the measurements of 2D conduit and reservoir strain values. Likewise, a negative correlation was observed for the passive atrial emptying fraction.

**Table 3:** Spearman correlations (p-value) of carotid-femoral pulse wave velocity with respective left atrial strain measurements

			Correlation (p-value)
**Two-dimensional echocardiogram**			
	LVEF (%)		0.11 (0.375)
	Indexed LA volume (ml/m²)		-0.07 (0.589)
**Strain 2D (%)**			
	Biplanar Reservoir		-0.27 (0.027)
	Biplanar Conduit		-0.29 (0.018)
	Biplanar Contraction		-0.12 (0.325)
	3D Volumes (mL)		
		Maximum	0.02 (0.879)
		Minimum	0.10 (0.422)
		Pre-A	0.12 (0.346)
**3D atrial emptying fraction (%)**			
	Total		-0.22 (0.078)
	Active		0.00 (0.974)
	Passive		-0.39 (0.002)
**Atrial distensibility index**			-0.22 (0.078)

LA: left atrial; LVEF: left ventricular ejection fraction.

## Discussion

### Arterial stiffness and atrial arrhythmia density

A recent review^15^ reported an association between arterial stiffness and the incidence of AF in different populations, even after cardioversion procedures and catheter ablation.^15^

In general, the origin of PAC is not too easy to be demonstrated by Holter monitoring. In the presence of PAC, as already known, the younger the patient (and absence of comorbidities), the greater the chance of the PAC origin in the right atrium, with the majority originating from the crista terminalis, while in older and hypertensive patients, this probability becomes more remote and the left atrium becomes the source of these PACs, especially with atrial stiffness or atrial overload caused by hypertension. We were careful to exclude single-focus atrial tachycardias and single-focus PACs by analyzing all Holter tracings.

A significant correlation was found between cfPWV and PACs in 24h. Also, we found a correlation of LV mass index with PACs in 24h and cfPWV; as a higher LV mass index can represent a confounding factor,^16,17^ it was decided to perform a generalized linear model with gamma distribution, adjusting for this variable. The correlation between cfPWV and PACs in 24h remained significant, indicating an independent association.

These findings may represent initial evidence in the literature of an association between arterial stiffness and the presence of atrial arrhythmias, still in the pre-fibrillatory phase, in hypertensive individuals, independently of the LV mass index. Although the correlation was considered weak, the fact that there is biological plausibility and that we found this correlation with AF strongly suggests that it was not an occasional finding.

### Left atrial function

In this study, we could demonstrate that the higher the PWV, the lower the conduit and reservoir strain values (2D), and the lower the atrial passive emptying fraction. These observations suggest that arterial stiffness may first affect left atrial reservoir and conduit functions and that dilation occurs later, as arterial stiffness increases, representing a late marker of atrial remodeling, as demonstrated by Yoshida et al.^18^ in a similar work with individuals with AF. It is important to point out that all the patients studied had LV mass index within normal values.

Our findings highlight the importance of assessing LA function in individuals with increased arterial stiffness, even in the absence of overt cardiovascular disease. LA strain, particularly reservoir and conduit strain, has been described as a more sensitive diagnostic marker of LV diastolic dysfunction than conventional tissue and spectral Doppler parameters.^19,20^

Brecht et al.^21^ demonstrated that impairment of reservoir and conduit strain was associated with diastolic dysfunction and that alteration of these two parameters preceded LA dilation and E/e’ elevation.^21^

### Clinical importance

Our data are important for understanding the pathophysiology of atrial cardiomyopathy, especially in the currently titled "pre-fibrillatory". Although the correlation between PWV and atrial ectopy density was weak, the fact that there are already studies demonstrating this association with AF reinforces the biological plausibility of our findings and agrees with the current literature. In addition, the importance of early detection of atrial remodeling in these patients is reinforced, even before LA dilation, paving the way for possible preventive strategies, in order to interrupt the cascade of atrial fibrosis, with the consequent development of AF.

In fact, the benefit of intervening in this phase before atrial dilation has already been demonstrated. Kokubu et al.^20^ reported a significant improvement in reservoir strain in hypertensive patients without LA dilation after two weeks of angiotensin-converting enzyme inhibitor therapy, whereas no improvement was observed in patients with LA enlargement.^20^

### Study limitations

Our study has some limitations. Ideally, the echocardiogram and PWV measurement should be performed simultaneously. However, as the echocardiographic technique does not allow simultaneous measurement of PWV, the tests could not be performed at the same time.

The low sample size can be explained because our hospital is a tertiary center, with a smaller proportion of patients with arterial hypertension without other comorbidities, since these patients are usually seen in the primary care setting in our country. Another possible limitation is the fact that it is not possible to exclude that all these patients had AF episodes excluded by other more precise tools, like implantable loop recorder. However this patient selection indeed reflects the "real world" clinical practice.

Finally, it is important to emphasize that this is a cross-sectional observational study. Therefore, it is not possible to establish a cause-and-effect relationship, and further prospective cohort studies are necessary in this field.

## Conclusion

Based on our observations, it was possible to conclude that arterial stiffness was associated with a higher burden of PACs, with worsening of LA function (conduit and reservoir), even before dilation of this cavity in these hypertensive elderly individuals (Central Illustration).

New studies with a larger number of patients are needed to corroborate our findings, since an association between arterial stiffness and a higher density of PACs was observed before the onset of AF.

Future clinical trials on arterial hypertension should analyze the impact of medications, not only on arterial stiffness but also on LA function and verify the benefit on decreasing LV mass index and improving atrial function, especially in those with frequent PACs.

## *Supplemental Materials

For additional information, please click here.
